# Supplementary material for: Rapeseed Domestication Affects the Diversity of Rhizosphere Microbiota
Source: Microorganisms. 2023 Mar 11;11(3):724. doi: 10.3390/microorganisms11030724 (PMC10056747; doi:10.3390/microorganisms11030724)
Supplement: Supplementary file 1 [file microorganisms-11-00724-s001.zip › Supplementary Figure.pdf]

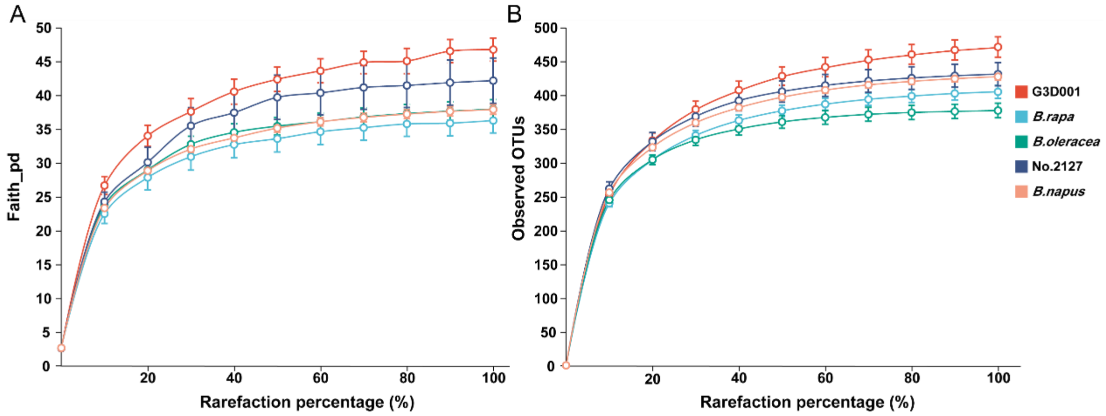

Figure S1. Rarefactions curves for observed OTUs and phylogenetic diversity metrics. With increasing sequencing depth, phylogenetic diversity (**A**) and observed OTUs (**B**) were gradually stabilized, indicating that the rarefaction curves of bacterial OTUs of the rhizosphere microbiota of *B. napus*, *B. olerac*ea, *B. rape*, and synthetic *B. napus* (G3D001 and No.2127) reached the saturation stage. Each vertical bar represents the standard error.

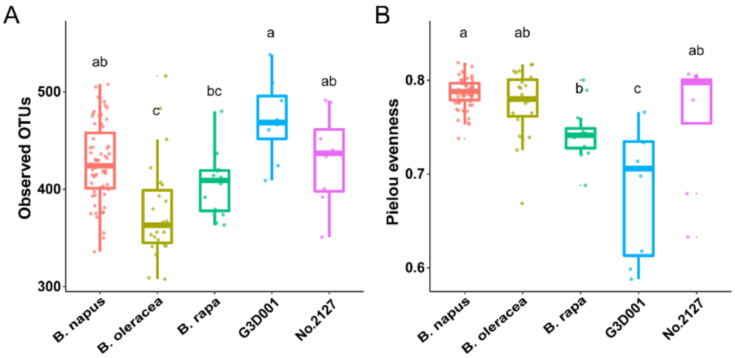

Figure S2. Alpha diversity in *B. napus*, *B. oleracea*, *B.rapa*, and synthetic *B. napus* G3D001 and No.2127 rhizosphere microbiota. Observed OTUs (**A**), Pielou evenness (**B**) of the rhizosphere microbiota of *B. napus*, *B. oleracea*, *B. rape*, and synthetic *B. napus.*

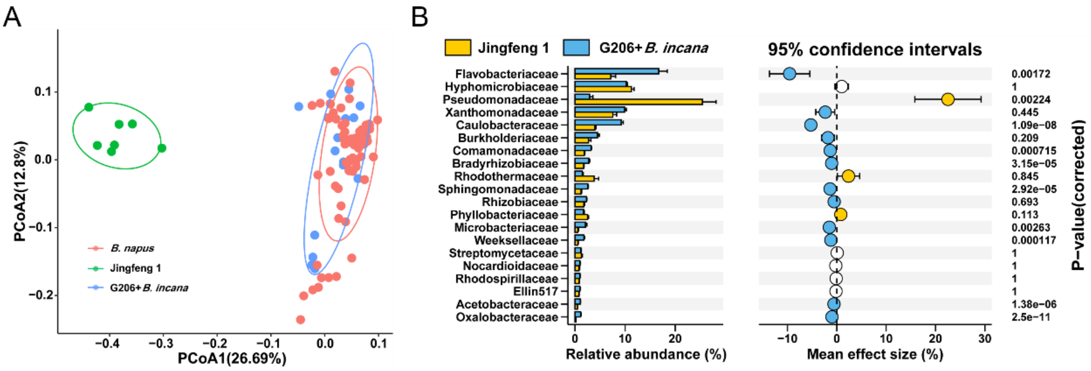

Figure S3. Differences between one accession of *B. oleracea* Jingfeng 1 and two other accessions of *B. oleracea* (G206 and *B. incana*). (**A**) Unconstrained PCoA based on Bray-Curtis distance demonstrated the rhizosphere microbiota of *B. napus*, *B. oleracea* Jingfeng 1, and *B. oleracea* (G206 and *B. incana*). (**B**) Differences between *B. oleracea* Jingfeng 1 and *B. oleracea* ((G206 and *B. incana*) at the family level.

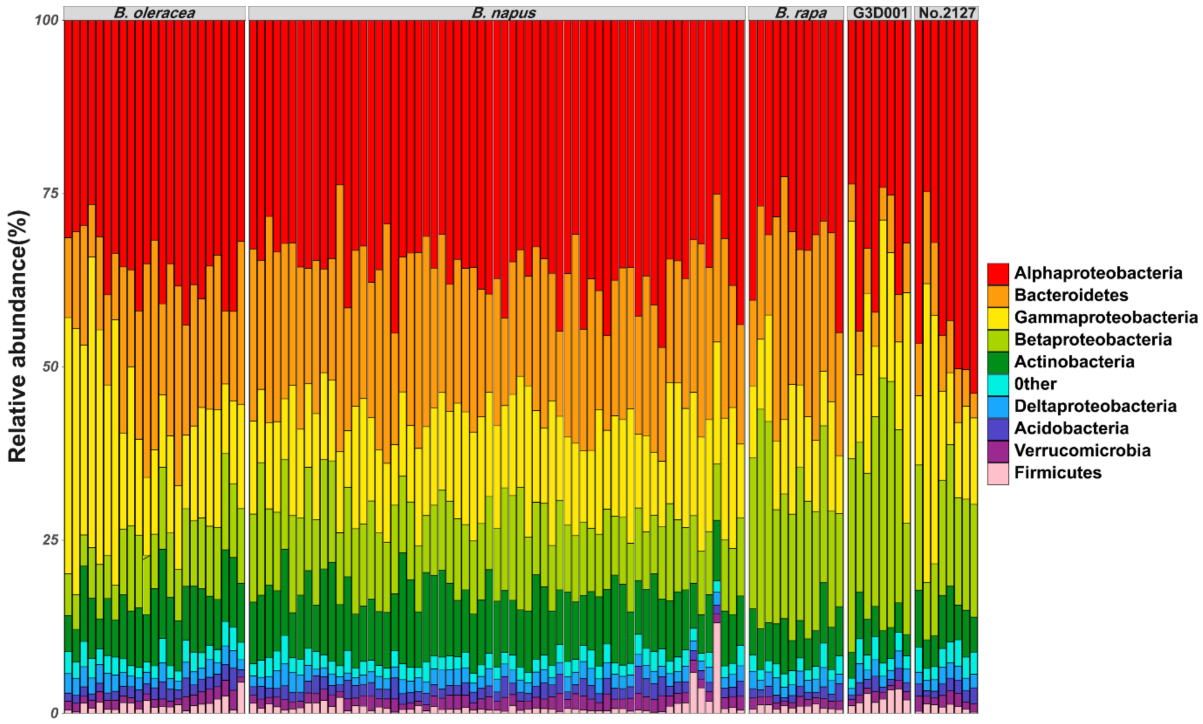

Figure S4. Composition of rhizosphere microbiota in all samples of *B. napus*, *B. oleracea*, *B. rape*, and synthetic *B. napus* G3D001 and No.2127. Proteobacteria is shown at the class level.

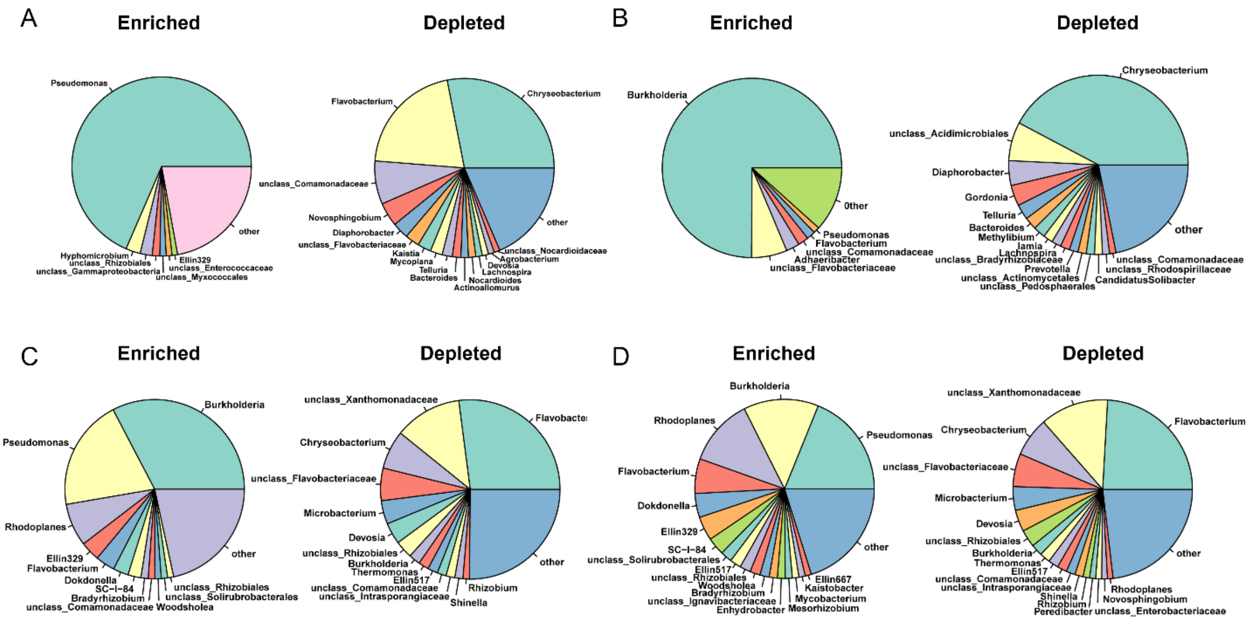

Figure S5. Abundance and taxonomy of enriched and depleted OTUs in *B. oleracea*, *B. rape,* and synthetic *B. napus* G3D001 and No.2127 relative to *B. napus*. Enrichment of OTUs was determined by using the relative abundance of *B. rape* (**A**), *B. oleracea* (**B**), G3D001 (**C**), and No.2127 (**D**), respectively. All depleted OTUs were determined by using the relative abundance of *B. napus.*

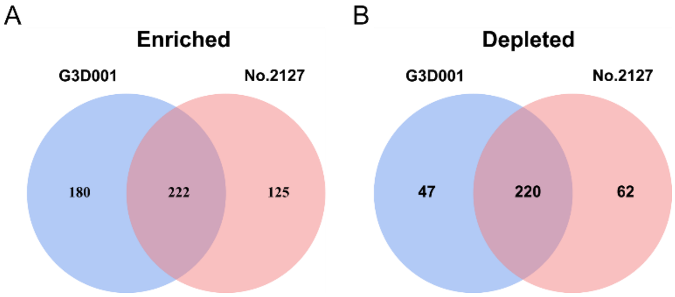

Figure S6. Co-enrichment and depletion of OTUs in synthetic *B. napus* G3D001 and No.2127 relative to *B. napus*. (**A**) Number of identical and different enriched OTUs in G3D001 and No.2127. (**B**) Number of identical and different depleted OTUs in G3D001 and No.2127.

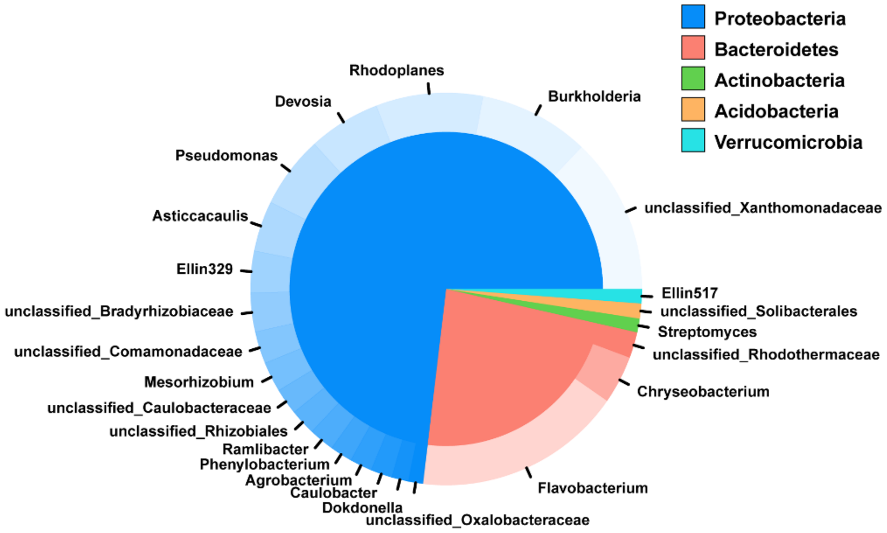

Figure S7. Rhizosphere core microbiota of *B. napus.* Different portions within the inner pie chart represent the bacterial phyla that are part of the *B. napus* core microbiota. The outer donut plot represents the genera that are part of the core, and each genus is assigned to the phylum to which it belongs. The sizes of the different pie and donut portions represent the contribution of each phylum/genus to the total relative abundance.

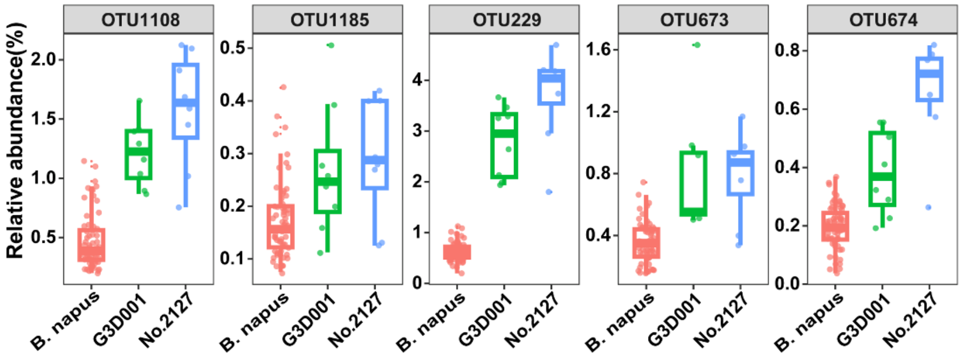

Figure S8. Relative abundance of OTUs that mainly affect the differences in nitrogen metabolism between *B. napus* and synthetic *B. napus* G3D001 and No.2127.
